# Artificial light at night (ALAN) negatively affects nest site occupancy but does not influence breeding success in two sympatric owl species

**DOI:** 10.1038/s41598-025-22044-9

**Published:** 2025-10-31

**Authors:** Erika Mátics, Miklós Laczi, Zoltán Schneider, Gyula Hoffmann, Róbert Mátics

**Affiliations:** 1 Doctoral School of Biology and Sport Biology, Faculty of Sciences, University of Pécs, Vasvári Pál u. 4., Pécs, 7622 Hungary; 2 Hungarian Nature Research Society, Vadvirág U. 5., Ajka, 8448 Hungary; 3 HUN-REN–ELTE–MTM Integrative Ecology Research Group, ELTE Eötvös Loránd University, Pázmány Péter Sétány 1/C, Budapest, 1117 Hungary; 4 Behavioural Ecology Group, Department of Systematic Zoology and Ecology, ELTE Eötvös Loránd University, Pázmány Péter Sétány 1/C, Budapest, 1117 Hungary; 5 The Barn Owl Foundation, Temesvári u. 8., Orosztony, 8744 Hungary; 6 Institute of Biology, Faculty of Sciences, University of Pécs, Ifjúság Útja 6., Pécs, 7624 Hungary; 7 Institute of Behavioural Sciences, Medical School, University of Pécs, Szigeti Út 12., Pécs, 7624 Hungary

**Keywords:** ALAN, Breeding site preference, Light pollution, Barn owl, Tawny owl, Reproduction

## Abstract

Artificial light at night (ALAN) negatively affects a broad range of animal species, with severe implications for conservation policy development and strategic planning globally. Birds are one of the most widely used ecological indicator groups in monitoring environmental changes. However, most studies examining the effects of ALAN are focused on diurnal bird species. It would therefore be necessary to study these effects in more detail on species with at least crepuscular or nocturnal activity, since they may be more vulnerable. We investigated the effects of illumination on the nest box occupancy of the western barn owl (*Tyto alba*; hereafter barn owl) and tawny owl (*Strix aluco*) in illuminated vs. unilluminated church towers and the reproductive output in nest boxes in these towers by comparing the numbers of eggs and chicks fledged. We found reduced breeding presence in illuminated towers in both species but no difference in reproduction parameters for either of the species. Our results underscore that light pollution has a negative consequence on the nest box occupancy of barn owls and tawny owls due to reducing breeding site suitability. This raises the threat that artificial light at night may hinder the conservation of such nocturnal bird species whose reproduction may be increasingly connected to human settlements in the future.

## Introduction

Light pollution and artificial light at night (ALAN) exerts major effects on a broad range of animal species^1,2^, affecting circadian rhythms^3–6^, hormone levels^7,8^, sleeping^9–11^, migration^12^, feeding behaviour^13,14^, predation pressure^15,16^, and its effects also appear at the community level^15,17–19^. Nocturnal species could be more vulnerable than diurnal ones^2^. Nocturnal vertebrates could show reduced activity when exposed to ALAN^20^. The impact of ALAN has also been demonstrated on small mammal populations^21–23^, which is particularly important given that this group constitutes a key prey source for owls, especially for the two species examined in our study. ALAN can either reduce^21,22^ or increase^23^ foraging behavior and activity levels in small mammals, which may influence their predators. In bats, ALAN has been shown to cause a range of effects, from altering foraging activity to roost abandonment, and may also indirectly negatively affect juvenile growth rates^24^.

Despite the continuous growth of research on ALAN, there are still significant gaps in our knowledge^25,26^. One of the gaps is that, in general, we know little about the effects on nocturnal owls^26^. Studies of crepuscular and nocturnal birds primarily focus on the feeding behaviour. There are numerous examples of nightjars^27–29^ and owls^30,31^ hunting around artificial nocturnal light sources, such as the glow of street lamps or illuminated buildings. At first, it might seem that ALAN could positively impact insectivorous birds by promoting the aggregation of their food sources, however this is not sufficiently supported. For example, Morelli et al.^32^ found a negative relationship between ALAN and insectivorous birds. ALAN can affect not only the foraging behavior of nocturnal species but also the composition of the diet^33^. In the case of the nightjar, artificial light at night (ALAN) may increase nocturnal flight activity^34^. Beyond all this, ALAN has a conservation interest, as it can directly contribute to the decline in populations of nocturnal avian species^35^. With regard to reproduction, it is known that ALAN can accelerate gonadal development in birds^36^ and it could influence the breeding ecology and biology of birds^37–39^. Some previous studies have revealed a negative correlation between owl occurrence and ALAN in urban areas^40,41^. However, our understanding of the effects of artificial light at night on the reproduction of nocturnal birds such as owls remains limited.

In Europe, the Barn Owl (*Tyto alba*) and the Tawny Owl (*Strix aluco*) are common species. The Barn Owl is strongly associated with human settlements, with its nesting primarily occurring in human-made structures^42^. Despite being primarily a woodland species, the urbanization of the Tawny Owl is well-documented^43–45^. As both species can nest in human settlements, where light pollution is most significant, they are good subjects for studying the effects of ALAN on occupancy and breeding patterns of nocturnal birds.

The aim of our research was to investigate the potential effect of building illumination on nest box occupancy and reproductive success of both species. We hypothesized that ALAN decreases the box occupancy in both species, as it likely represents a disturbance for these nocturnal birds. Previous studies have shown negative correlation between tawny owl and illuminated areas^26,41^. Consistent with these findings, our hypothesis was that the Tawny Owl is likely to be more severely affected, as it is originally a woodland species that is less habituated to light pollution compared to the Barn Owl, which is already associated with human-made structures. We also predicted that breedings exposed to ALAN will exhibit lower reproductive success.

## Materials and methods

### Study area

**Fig. 1:**
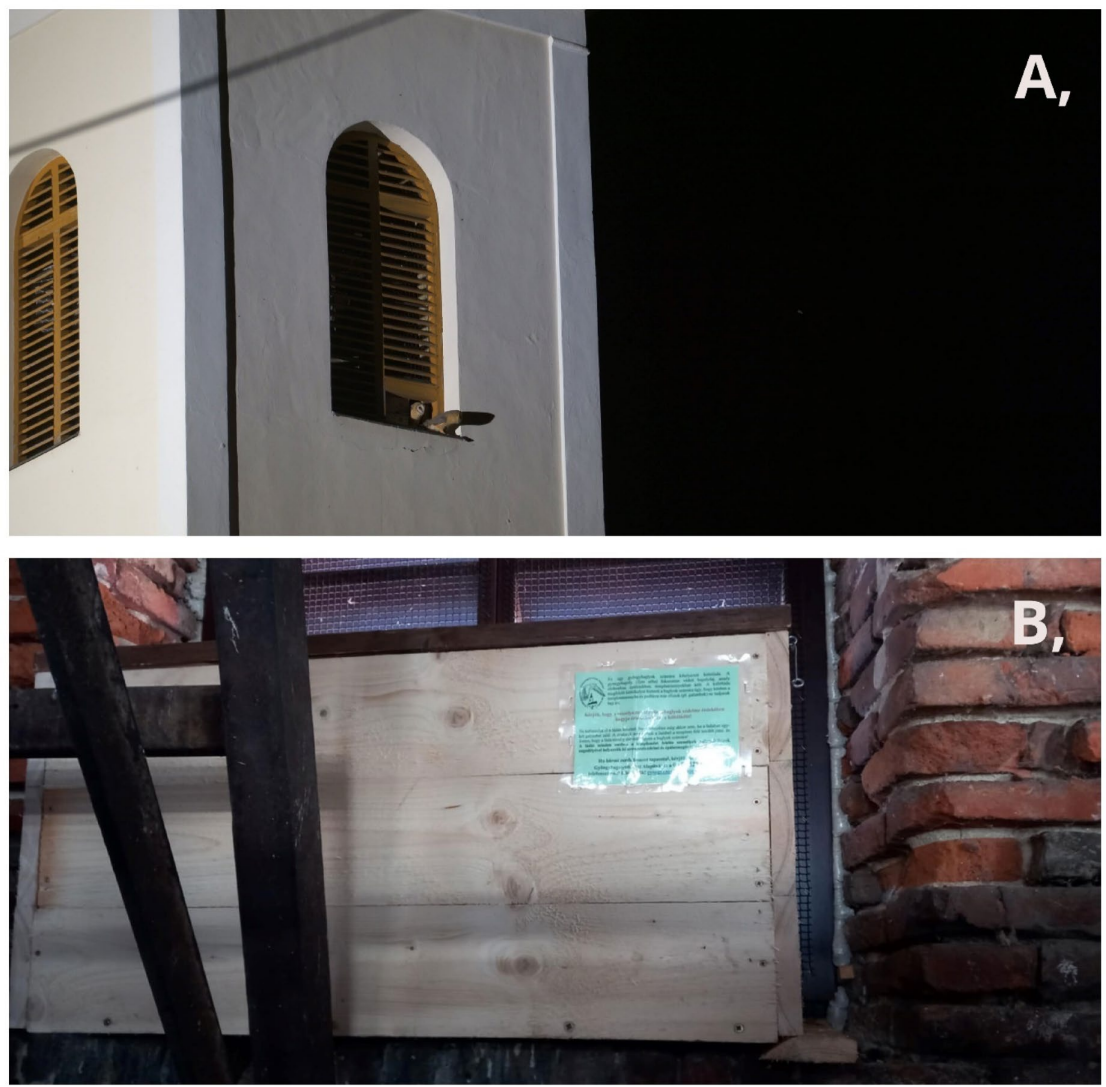
**A** An illuminated church tower currently occupied by barn owls. **B** Location of the nest box positioned behind the church window. The birds can only access the nest box, as it is closed off toward the interior of the building.

The study was carried out in Baranya County (4430 km^2^; 46°04′ N, 18°14′ E), located in southwestern Hungary. The rural county is characterized by small settlements surrounded by natural habitats and agricultural lands, rather than larger cities. In 96% of the 301 settlements, at least one church tower or chapel can be found. A total of 163 exterior nest boxes were placed in different buildings, of which 95% were church or chapel towers (the remaining 5% was placed in farm buildings or in the attics of large buildings), progressively from 1988 to 2019. The nest box dimensions were 100 × 50 × 50 cm in general but could differ according to the availability of space in the towers. Each box was placed behind the tower’s window with a 15 cm*15 cm hole as the entrance (Fig. 1). The artificial nest boxes were installed across 150 different settlements^46^. For this study, we used data only from nest boxes placed in church towers.

### Data collection

Breeding data was gathered by volunteers (50–60 people) of the Baranya County Group of BirdLife Hungary based on a strict protocol^46^. We recorded the illumination state of each church along with the year of the installation of the illumination. We considered a tower unilluminated if it was not illuminated at all during the year or if it was illuminated only twice a year (i.e., at Christmas and Easter). In the other cases the towers were connected to the public lighting system, that means these buildings were illuminated every night. These were considered illuminated. As it was not possible to measure the light intensity of the towers’ illumination, we considered only their presence or absence in the following analysis. During the illumination of individual buildings, the property maintainers applied various methods and types of lighting. However, the nest boxes were always placed behind windows that had only a single entrance hole for the birds to fly in, and the boxes were closed on the side facing the interior of the building, preventing owls from entering the building itself. Since the boxes are connected to the outside environment only through the entrance hole, it can be stated that there is no direct lighting inside the box where breeding takes place.

For nest box occupancy, we used a presence/absence variable, and nest box was considered occupied if a clutch was found in it. For the analyses, we used the following breeding variables: clutch size (i.e., the number of eggs laid), fledgling number, and laying date (compared to the yearly median). In barn owls, second broods are known to be common in years with high food availability^42,47–49^, whereas in tawny owls this breeding behavior is very rare^50^. Importantly, only the first broods were used from a given nesting location. Furthermore, we excluded the cases of the respective variables where the laying date was uncertain, the clutch was not completed (due to interruption, e.g., predation, nest desertion), or the number of fledglings was uncertain (due to predation on owlets or nest abandonment by the parents). Accordingly, for clutch size and fledging number, we use data where the value was larger than 0.

We aimed to investigate if there were differences in the presence of barn owl or tawny owl breeding in the mirror of the illumination state of the church towers. The towers were only considered from the year when the given owl species first bred there. This is important because it’s possible that, after placing the boxes, there may be no owls in the area for years. (Our unpublished results suggested that the number of years from the date of nest box establishment to the date of first occupancy was not related to the illumination status of the tower.) According to this, for the presence/absence we had 952/1207, and 249/359 cases in the barn owl (1988–2019) and the tawny owl (1992–2019), from 122 locations in sum (meaning 119 and 45 locations for the barn owl and the tawny owl, respectively, with 42 overlapping locations between the two species).

All methods were carried out in accordance with relevant guidelines and regulations (Pest County Government Office, Department of Environmental Protection and Nature Conservation, Permit Nr. PE-KTF/97-13/2017).

### Statistical methods

For analysing nest box occupancy, we applied generalized linear mixed-effects models (GLMMs) with binomial error distribution, logit link function, and Satterthwaite estimation for degrees of freedom. The presence/absence was the binary response variable, and the illumination state was the fixed factor. We also controlled for location by entering location identity as a random effect to account for potential location-specific variations (such as microhabitat or prey density) without confounding the effect of illumination, because location was not 1:1 with the nest box, as multiple nest boxes/towers were used across different years, hence locations were represented multiple times.

We used Chi-square tests to assess if there was a difference between the two species with respect to the association of presence or absence in unilluminated and also illuminated towers.

Additionally, we also conducted Wilcoxon matched pair tests in order to evaluate the potential changes with respect to the scores for presence in towers of which illumination states were altered since the owl’s first presence there. Hence, we calculated average presence scores for the relevant time periods in each tower (i.e., N_years with presence_/N_total years_) separately for the illumination states. Typically, the direction of the status change was that the towers went from unilluminated to illuminated (with the exception of one case where it was subsequently darkened again; this data series was not used here). In addition, only sites were included in the analysis where the ratio between illuminated and unilluminated years was a maximum of 3:1 or a minimum of 1:3. Accordingly, our samples consisted of 21 and 7 paired observations in the barn owl and the tawny owl, respectively, and the relevant locations were represented by one data point per group.

We investigated if reproduction success in the first broods was associated with the illumination state (two levels: 0 = not illuminated, 1 = illuminated) of the towers. In order to do this, we conducted linear mixed models (LMMs) with normal error, identity link function, and Satterthwaite estimation for degrees of freedom. We used one of the reproductive variables (clutch size, fledging number) as a response variable, the illumination status of the church towers, and the laying date as fixed effects. Additionally, we controlled for year and location using these as random effects. We entered laying date as a fixed effect to control for the potential effects of the date on reproduction output. For fixed effects, we applied backward stepwise model simplification^51^. (However, with regard to the illumination state, we kept qualitatively the same results if we did not consider the laying date.) According to QQ-plots, model residuals were normally distributed.

We analyzed the data from the two owl species separately in all cases.

The analyses were performed in R 4.2.1^52^. For the GLMMs and LMMs, we applied the glmer() and the lmer() functions, respectively, from the ’lme4’ package^53^, and the Anova () function from the ‘car’ package^54^. We obtained the conditional and marginal R^2^ values using the r.squaredGLMM() function from ’MuMin’ package^55^. The Chi-square tests were conducted using the chisq.test() function in R, the Wilcoxon matched pair tests were conducted using the wilcox,test() function.

The primary data supporting the results of this study are available at the Figshare digital repository^56^.

## Results

**Fig. 2:**
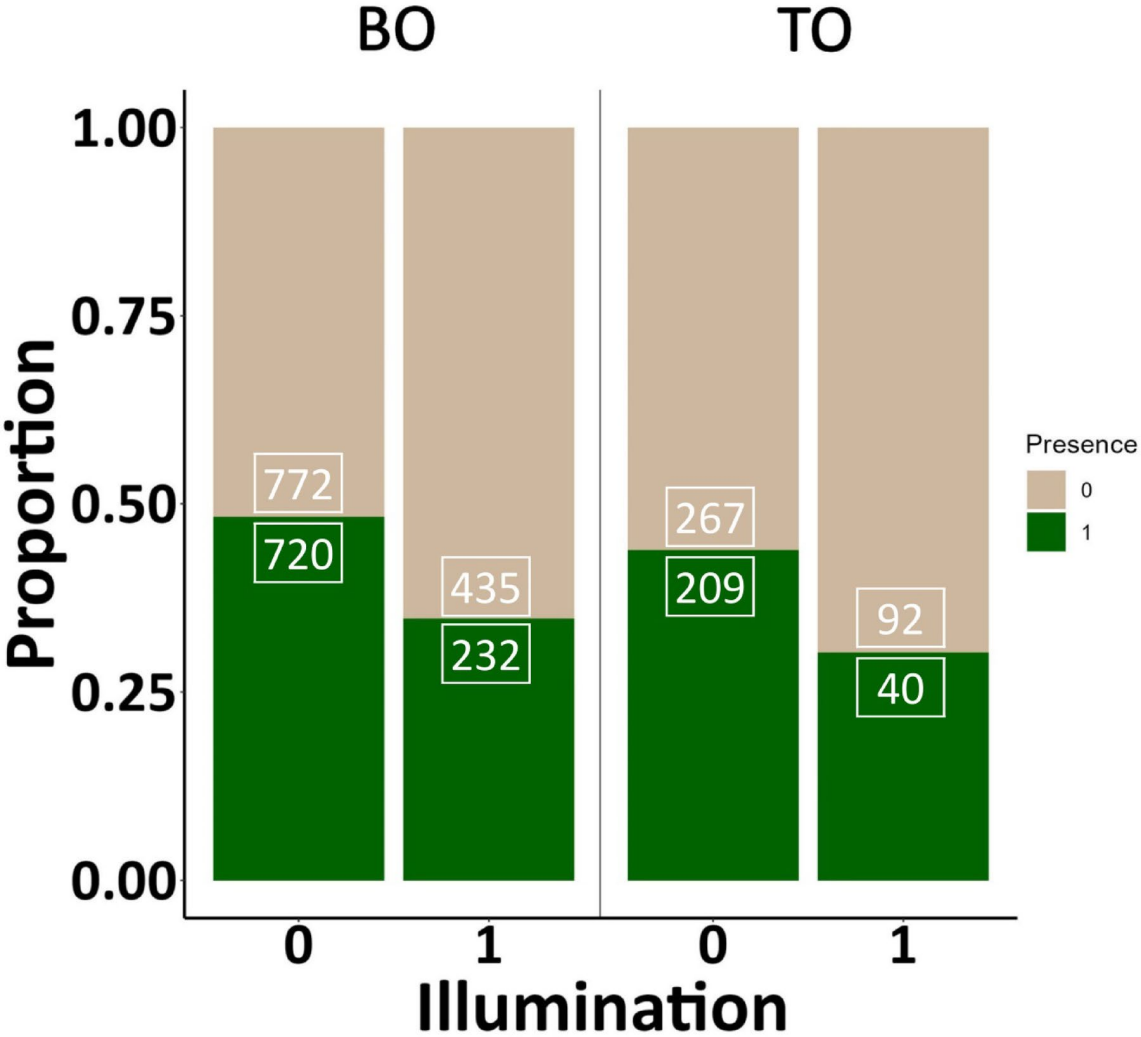
Proportions of the presence (drak green) and absence (light brown) of the barn owl (BO) and tawny owl (TO) in unilluminated (0) and illuminated (1) church towers. The numbers in the boxes refer to the sample size of the respective group.

The GLMMs revealed in both species that the nest box occupancy was significantly higher in church towers with no illumination (see details on estimate(SE), Wald χ2, significance, conditional and marginal R^2^ in Table 1 and Fig. 2).

**Table 1:** Association between presence of breeding barn owl and tawny owl in connection with the unilluminated versus illuminated state of church towers.

Species	Explanatory variable	Estimate(SE)	Wald χ2(df)		R^2^m	R^2^c
Barn owl	(Intercept)	0.04(0.13)	0.08(1)			
	Illumination	− 0.74(0.17)	18.33(1)	***	0.03	0.31
Tawny owl	(Intercept)	− 0.09(0.22)	0.16(1)			
	Illumination	− 0.86(0.35)	6.14(1)	*	0.03	0.32

According to the results, the presence was negatively associated with the illuminated state of the towers in both species. We provided marginal R^2^ (R^2^m) and conditional R^2^ (R^2^c) for fixed effects retained in the final model after backward stepwise model simplification.

*P < 0.05, **P < 0.01, ***P < 0.001. We applied generalized linear models with binomial error distribution and logit link function.

According to the Chi-square tests, the two species showed no differences from each other with respect to presence/absence (unilluminated towers: χ2 = 2.57, df = 1, *P* = 0.10; illuminated towers: χ2 = 0.80, df = 1, *P* = 0.37).

The Wilcoxon matched pair tests revealed marginal effects on the average scores of presence (barn owl: W = 170, P = 0.060; tawny owl: W = 25, *P* = 0.078), suggesting a decreasing trend in presence in formerly unilluminated towers that have become illuminated. The median difference between ’pre-treatment’ and ’post-treatment’ scores was -0.29 and -0.53 for the barn owl and the tawny owl, respectively.

The investigated reproduction parameters were not associated with the illumination state of the towers in either species, and the laying was the only predictor that was slightly correlated with clutch size and fledging number (see details on estimate(SE), Wald χ2, significance, conditional and marginal R^2^ in Table 2, and the descriptive statistics of reproductive variables in Table 3).

**Table 2:** Relationships of reproductive parameters with the illumination state of church towers in the barn owl and tawny owl.

Species	Response variable	Explanatory variable	Estimate(SE)	Wald χ2(df)		R^2^m	R^2^c
Barn owl	Clutch size	(Intercept)	6.80(0.13)	2886.67(1)	***		
		**Laying date**	0.01(0.00)	16.94(1)	***	0.02	0.21
		Illumination	− 0.18(0.14)	1.64(1)			
	Fledgling number	(Intercept)	4.72(0.15)	951.59(1)	***		
		**Laying date**	0.01(0.00)	15.82(1)	***	0.02	0.23
		Illumination	− 0.01(0.15)	0.01(1)			
Tawny owl	Clutch size	(Intercept)	4.40(0.16)	780.15(1)	***		
		Laying date	− 0.01(0.01)	2.52(1)			
		Illumination	− 0.28(0.29)	0.34(1)			
Tawny owl	Fledgling number	(Intercept)	3.16(0.14)	542.21(1)	***		
		**Laying date**	− 0.01(0.01)	3.99(1)	*	0.02	0.29
		Illumination	0.08(0.26)	0.08(1)			

We provided marginal R^2^ (R^2^m) and conditional R^2^ (R^2^c) for fixed effects retained in the final model after backward stepwise model simplification.

*P < 0.05, **P < 0.01, ***P < 0.001. Terms retained in the final model are highlighted in bold.

**Table 3:** Descriptive statistics of reproductive variables between two groups of the illumination state of church towers in the barn owl and tawny owl.

Species	Illumination state	Reproduction variable	Mean	SD
Barn owl	Illuminated	Clutch size	6.94	1.47
		Fledging number	4.86	1.60
	Not illuminated	Clutch size	6.79	1.46
		Fledging number	4.88	1.61
Tawny owl	Illuminated	Clutch size	4.43	1.07
		Fledging number	3.22	1.17
	Not illuminated	Clutch size	3.89	1.02
		Fledging number	3.07	1.04

## Discussion

Light pollution poses a serious threat to numerous species^1,2,57^ and our study indicates that also owls are negatively affected by ALAN. Our results revealed that nestbox occupancy probability was higher in unilluminated than illuminated towers, as a significantly lower number of breedings occurred in illuminated towers compared to unilluminated ones. However, illumination did not reduce the reproductive output (measured in clutch size and fledgling number) in either species.

Both bats and owls are nocturnal animals and often prefer the same buildings as resting and breeding sites^58,59^. Based on this, it can be assumed that the illumination of these buildings may have a similar impact on both groups. For bats, lighting of churches is known to directly lead to the abandonment of these buildings as roost sites^60,61^. In our study, in the case of towers previously unlit but later illuminated, we observed a marginal, not significant effect of illumination on the reduction in nesting events following the lighting. The fact that the abandonment of buildings as nesting sites due to lighting has less of an impact on these two owl species than on bats may stem from the possibility that these species are perhaps less sensitive to the quality of nesting sites than bats. It is known that the barn owl originally nested in hollow trees, but with their disappearance, it adapted to human-made structures. Today, it nests not only in churches and farm buildings but also in the attics of family houses, water towers, and even in loess cliffs^42,62^. The Tawny Owl, on the other hand, can nest on the ground, in abandoned twig nests, or even in haystacks, in addition to buildings and natural tree cavities^63^. Our hypothesis was that the effects of ALAN would impact the tawny owl more strongly than the barn owl, but we did not find such a difference. This may be explained by the fact that, although the tawny owl is originally a woodland species, it shows a high degree of urbanisation^43–45^, which likely enables it to tolerate light-polluted areas to some extent. However other studies^26,41^ demonstrated a negative association between the presence of tawny owls and ALAN in urban environments. One possible explanation is that the tawny owl shows a tendency toward urbanization but avoids areas with excessive light pollution. Illumination may affect owls not only directly, but also indirectly by influencing prey availability. Several studies have shown that ALAN can have an effect on small mammals^21–23^, for example by reducing their activity or foraging behavior^21,22^.

For crepuscular and nocturnal birds, we have little knowledge about the effects of ALAN on their breeding biology. In the case of the common nighthawk (*Chordeiles minor*) and common poorwill (*Phalaenoptilus nuttallii*), it is likely that the birds avoid areas polluted by ALAN due to the higher nest predation risk associated with it^64^. Our knowledge of the effects of ALAN on bird breeding biology is primarily related to diurnal bird species. These effects can be either positive^37^ or negative^39^. ALAN can influence the begging time of nestlings^37^, extend the time that parents spend bringing food^37,65^, advance the laying date^38^, and reduce the weight gain of the nestlings^39^. In our study, we did not find any evidence that ALAN affected the breeding success of the two owl species. The owls do not nest freely in any part of the building but rather in enclosed artificial nest boxes placed behind the windows. Since these boxes are closed (open only through the entry hole), light pollution likely has a reduced impact inside, exposing the eggs or chicks to only lower-intensity light stress. It is also possible that illumination may have different, potentially even positive, effects on owls. For example, by facilitating hunting which could explain why we do not observe a reduction in reproductive success. However, the fact that the building itself is illuminated may still disturb the birds in their choice of nesting site, even though the box remains quite dark. Supporting this, data on the presence-absence of nesting show a significantly negative effect when the building is illuminated.

We are aware of the limitations of our study: although we did not observe a significant effect on clutch size or fledging number, the effects of ALAN may manifest, for example, in the form of altered immune function^66^ or reduced body mass^39^.

In several regions, barn owl populations have been displaced from churches primarily due to renovations^67,68^, yet in Hungary, the majority of known breeding sites are still found in church towers^46,62,68^. Given the declining population trends of the barn owl in multiple regions^42,69,70^, church illumination adds an additional negative impact on this already vulnerable species.

Our results elucidate the broader ecological impacts of light pollution on nocturnal birds, contributing to our understanding of how ALAN influences life history traits and conservation outcomes for these species.

## Data Availability

The primary data supporting the results of this study are available at the Figshare digital repository10.6084/m9.figshare.27930849.v1.
